# Supplementary material for: Predicting Egg Passage Adaptations to Design Better Vaccines for the H3N2 Influenza Virus
Source: Viruses. 2022 Sep 17;14(9):2065. doi: 10.3390/v14092065 (PMC9501976; doi:10.3390/v14092065)
Supplement: Supplementary file 1 [file viruses-14-02065-s001.zip › Supplementary Figure S1.pdf]

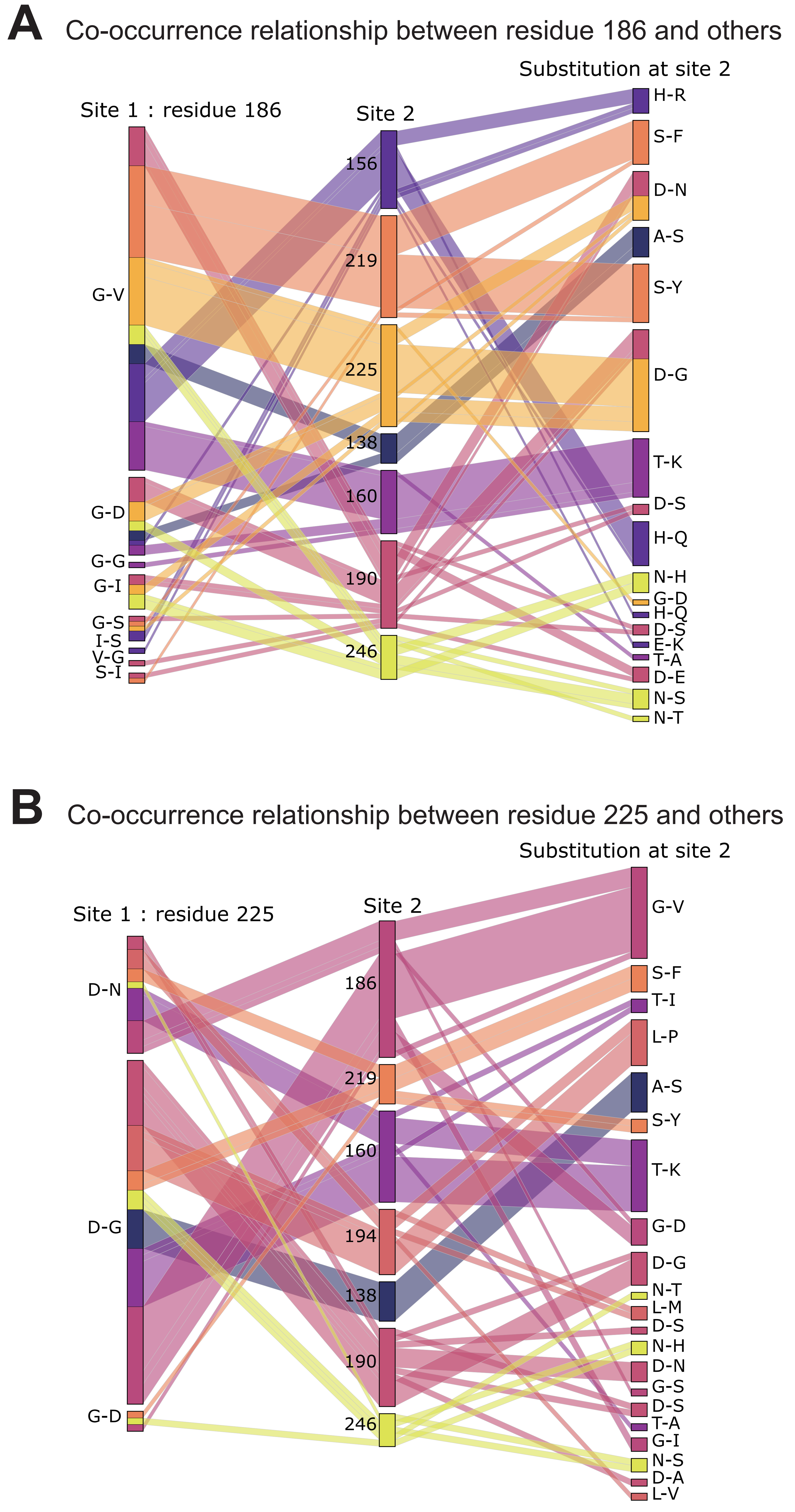
A
Co-occurrence relationship between residue 186 and others
Substitution at site 2
Site 1 : residue 186
Site 2
G-V
G-D
G-G
G-I
G-S
I-S
V-G
S-I
156
219
225
138
160
190
246
H-R
S-F
D-N
A-S
S-Y
D-G
T-K
D-S
H-Q
N-H
G-D
H-Q
D-S
E-K
T-A
D-E
N-S
N-T
B
Co-occurrence relationship between residue 225 and others
Substitution at site 2
Site 1 : residue 225
Site 2
D-N
D-G
G-D
186
219
160
194
138
190
246
G-V
S-F
T-I
L-P
A-S
S-Y
T-K
G-D
D-G
N-T
L-M
D-S
N-H
D-N
G-S
D-S
T-A
G-I
N-S
D-A
L-V
