# Supplementary material for: Predicting Egg Passage Adaptations to Design Better Vaccines for the H3N2 Influenza Virus
Source: Viruses. 2022 Sep 17;14(9):2065. doi: 10.3390/v14092065 (PMC9501976; doi:10.3390/v14092065)

The work flow of the predictive model

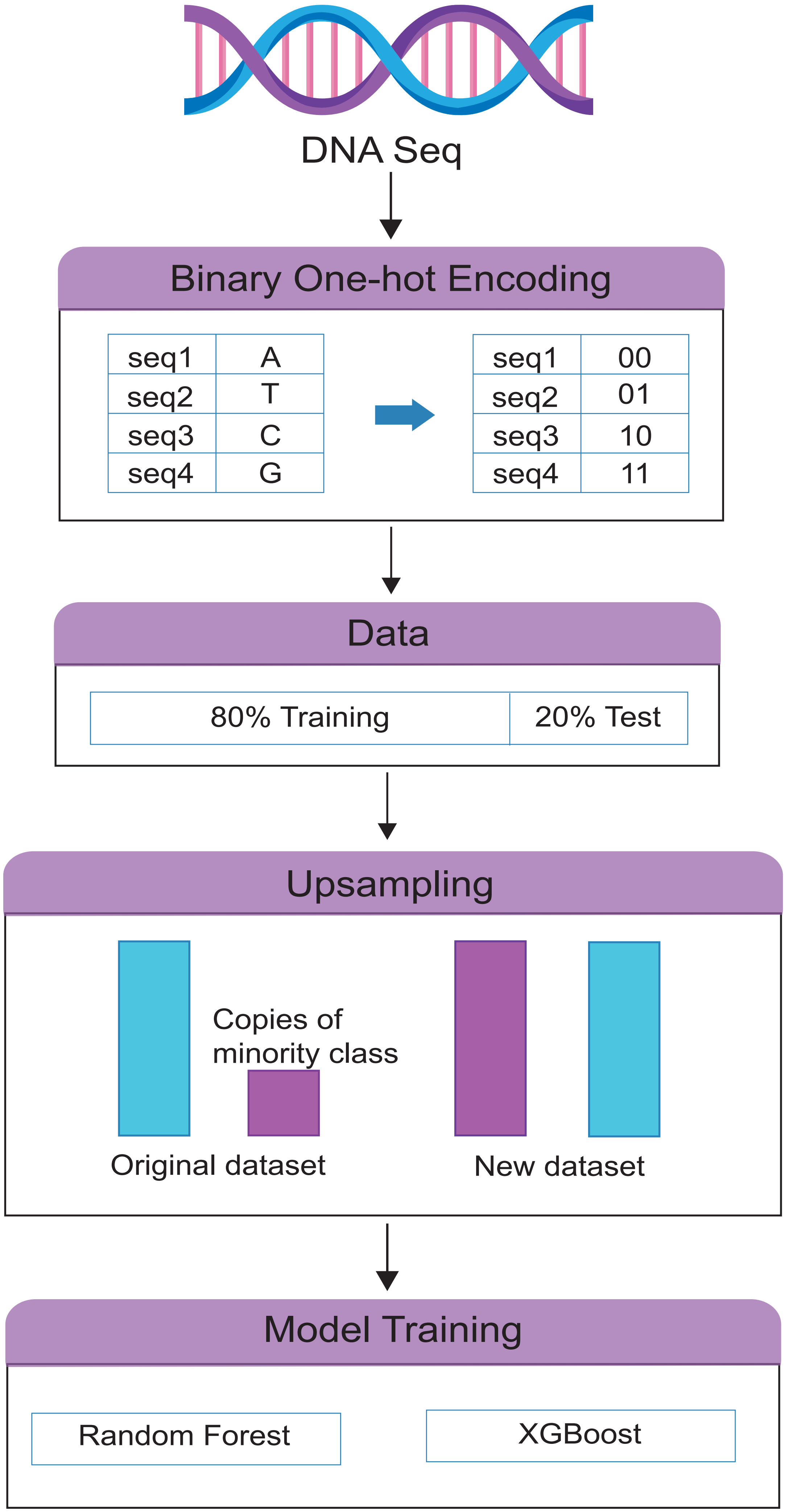

Supplement: Supplementary file 1 [file viruses-14-02065-s001.zip › Supplementary Figure S5.pdf]
