# Supplementary material for: Predicting Egg Passage Adaptations to Design Better Vaccines for the H3N2 Influenza Virus
Source: Viruses. 2022 Sep 17;14(9):2065. doi: 10.3390/v14092065 (PMC9501976; doi:10.3390/v14092065)
Supplement: Supplementary file 1 [file viruses-14-02065-s001.zip › Supplementary_Figure_legends.docx]

**Supplementary Figure S1. Examples of co-occurrence relationships between substitutions at different amino acid sites.**

A) For residue 186, The first column shows the substitution type at the first amino acid site, the second column indicates the second amino acid site, and the third column plots the type of substitutions occurring at the second amino acid site. Each line represents one egg branch. B) The same plot for residue 225.

**Supplementary Figure S2. Boxplot for the ES scores at the 18 residues.**

**Supplementary Figure S3. PCA of 89,853 sequences based on ES at 18 residues.**

A) Visualization of 2D projection of sequences to the top 2 PCs. The inset figure highlighted the egg strains. B) PC loadings of the 18 residues.

**Supplementary Figure S4. Vaccine efficacy and frequencies of different substitutions.**

Vaccine efficacy and frequencies of substitution in major codons

**Supplementary Figure S5. The flow chart of the machine learning model**

A illustration of the predictive model starting from DNA sequences to binary one-hot sequencing, to upsampling and modeling training. We used 80% of the data (n=688) to train the model and used the remaining 20% dataset to test the model.
