# Supplementary figures and images for: Predicting Egg Passage Adaptations to Design Better Vaccines for the H3N2 Influenza Virus
Source: Viruses. 2022 Sep 17;14(9):2065. doi: 10.3390/v14092065 (PMC9501976; doi:10.3390/v14092065)

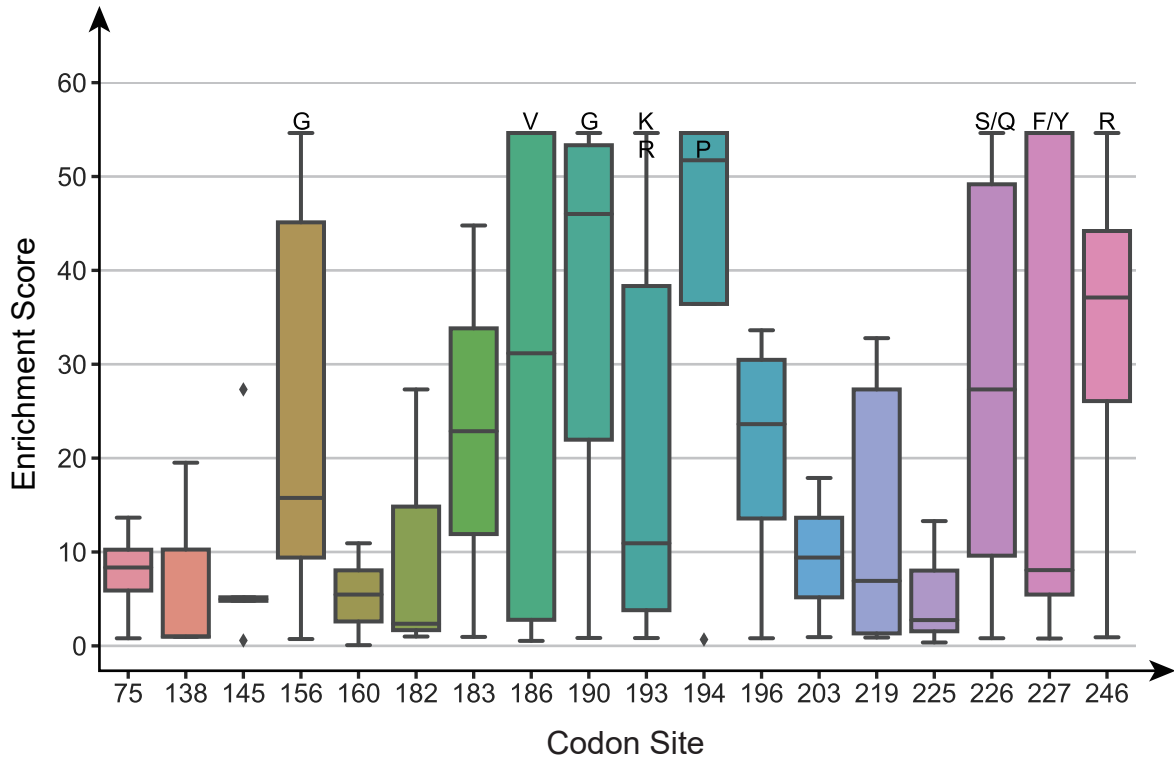

Supplement: Supplementary file 1 [file viruses-14-02065-s001.zip › Supplementary Figure S2.pdf]

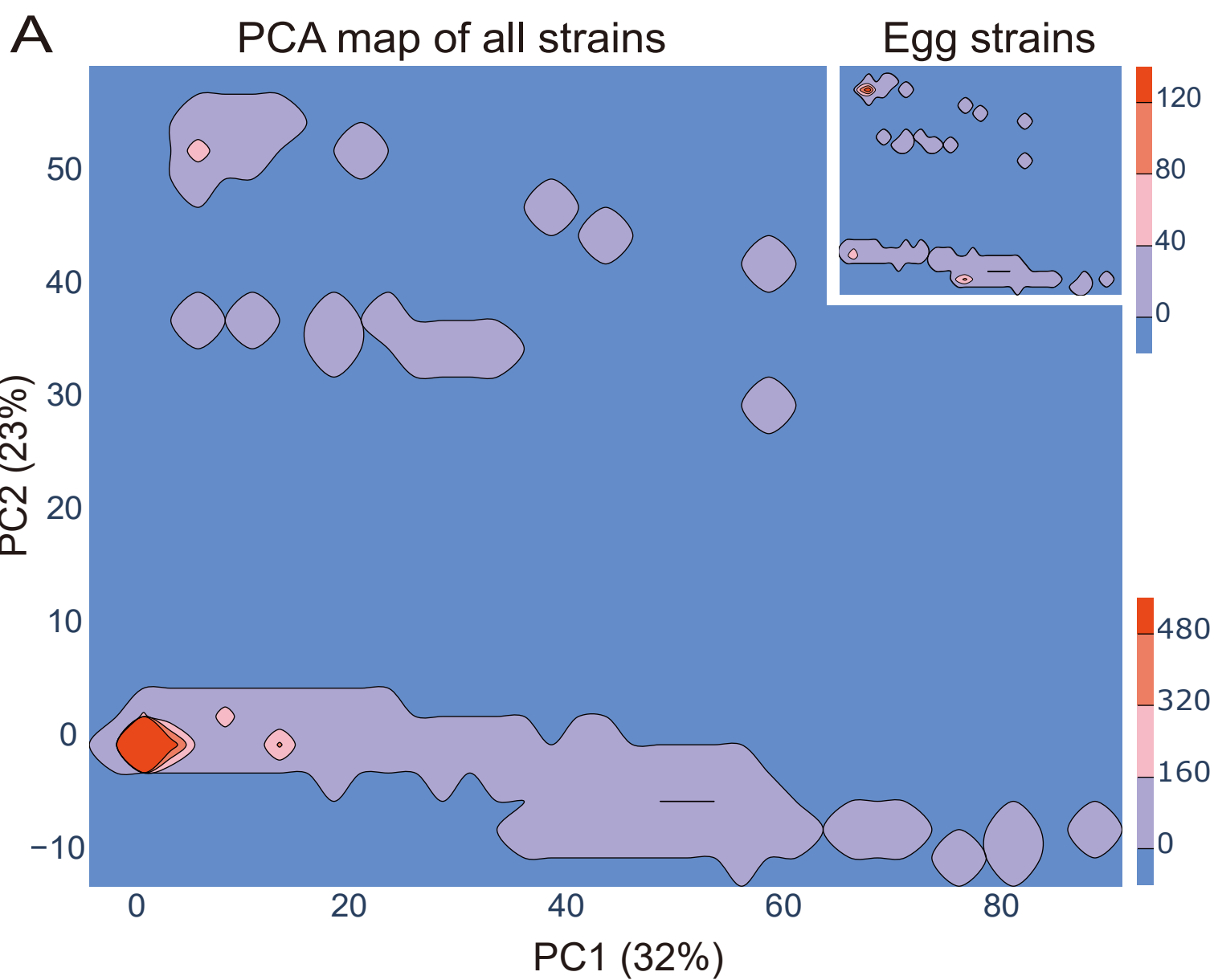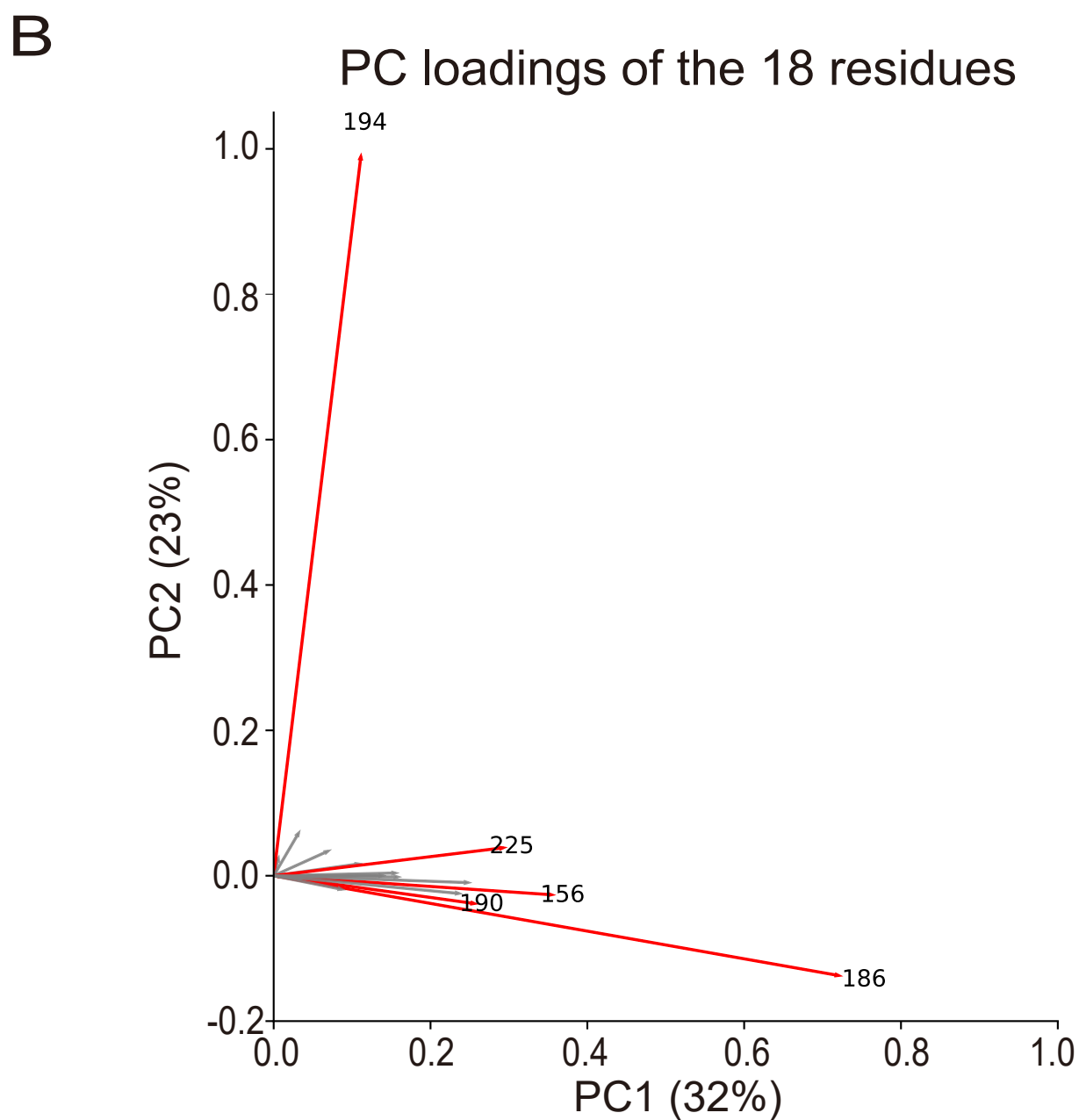

Supplement: Supplementary file 1 [file viruses-14-02065-s001.zip › Supplementary Figure S3.pdf]

# Vaccine efficacy and frequency of different substitutions

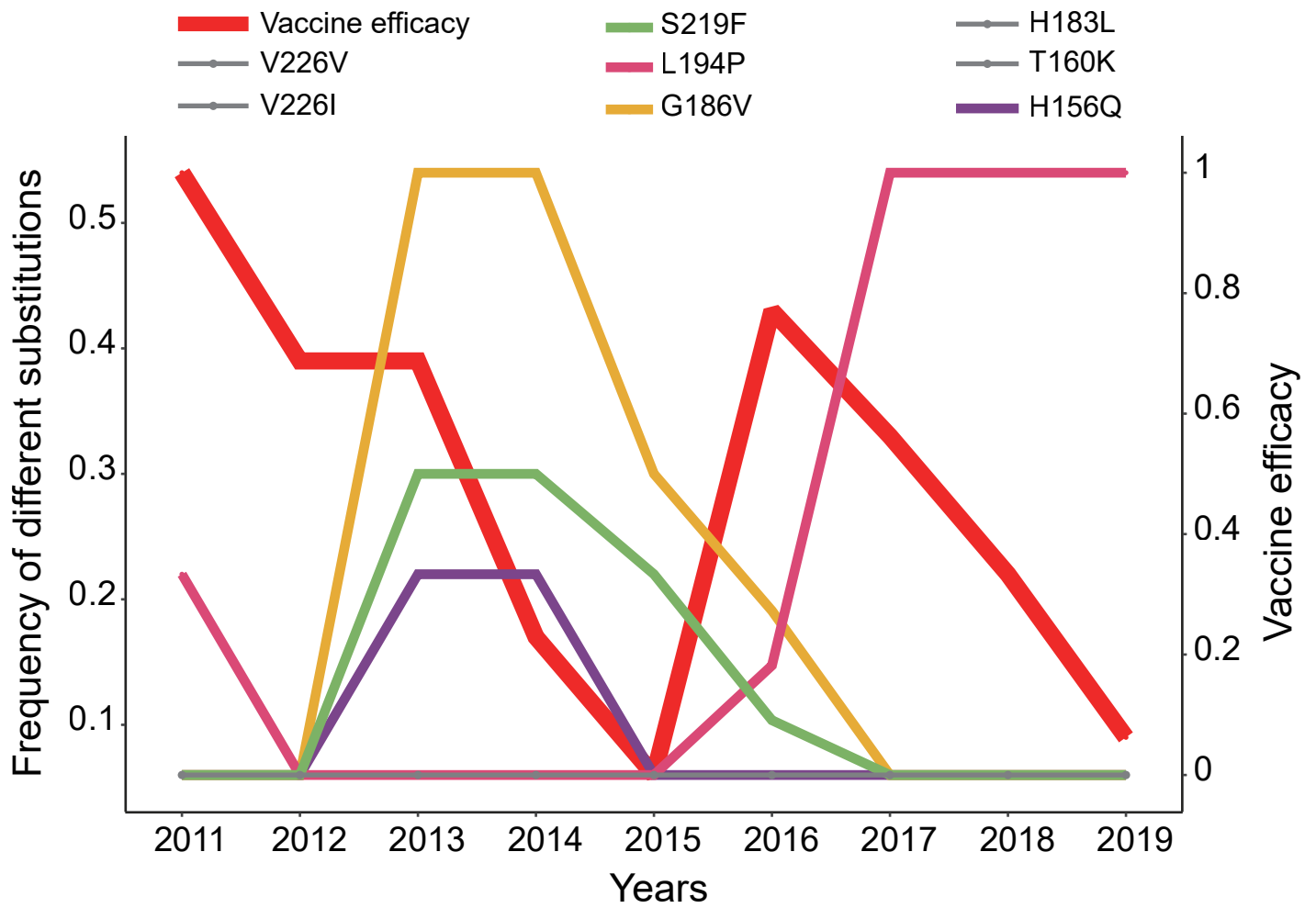

Supplement: Supplementary file 1 [file viruses-14-02065-s001.zip › Supplementary Figure S4.pdf]
